# The gut microbiota-enteric nervous system axis: from bidirectional programming to precision therapeutics in digestive diseases

**DOI:** 10.3389/fcimb.2026.1852596

**Published:** 2026-06-10

**Authors:** Huimin Li, Ao Ye, Jieyu Song, Ying Yang, Jun Li

**Affiliations:** 1 Department of Clinical Medicine, Chengdu Medical College, Chengdu, Sichuan, China; 2 Department of Gastroenterology, First Affiliated Hospital of Chengdu Medical College, Chengdu, Sichuan, China

**Keywords:** colorectal cancer, enteric nervous system, gut microbiota, inflammatory bowel disease, irritable bowel syndrome

## Abstract

The bidirectional communication between the gut microbiota (GM) and the enteric nervous system (ENS) is fundamental for gastrointestinal homeostasis. This review dissects the intricate GM–ENS dialogue, emphasizing its transition from a developmental programmer in early life to a sustained regulator of neural plasticity in adulthood. We synthesize the core signaling mechanisms—microbial metabolites, neuroactive substances, and immune mediators—that orchestrate ENS activity. We further propose a multi−timescale model (rapid neuropod/bioelectric, intermediate metabolite, slow immune) with differential relevance to irritable bowel syndrome (IBS) (fast distortions) versus inflammatory bowel disease(IBD)(slow amplification). Critically, we delineate how this axis adopts distinct pathophysiological roles across major digestive disorders: acting as a “signal distorter” in IBS, an “inflammation amplifier” in IBD, and a “tumor accomplice” in colorectal cancer (CRC). By integrating these mechanistic insights with emerging therapeutic paradigms (e.g., precision biomarkers, synthetic microbial consortia, postbiotics), we position the GM–ENS axis as a central hub for understanding disease pathogenesis and a promising framework for developing next-generation precision medicine strategies.

## 1 Introduction

Functional gastrointestinal disorders affect approximately 40% of the global population, with IBS representing a significant contributor to this burden due to its complex pathophysiology and substantial impact on quality of life (Sperber et al., 2021). According to the latest meta-analyses employing Rome IV criteria, the global prevalence of IBS ranges from 13.28% (in probability-sampling studies) to 17.14% (overall), with a notably higher prevalence in women (Ballena-Caicedo et al., 2025). Concurrently, IBD has evolved from a Western-centric condition into a global health challenge. Epidemiological models project that within the next decade, more than 1% of the population in early-industrialized countries will be living with IBD, while newly industrialized nations are experiencing rapidly accelerating incidence rates (Kaplan, 2025). CRC further compounds this burden, ranking as the second leading cause of cancer-related mortality worldwide with approximately 2 million new cases and nearly 1 million deaths annually (Chen et al., 2025). Of particular concern, early-onset CRC (occurring in individuals under 50 years) is increasing across both high-income and emerging regions, signaling an urgent public health challenge (Mauri et al., 2019).

Despite substantial advances in understanding the pathophysiology of these disorders, current therapeutic approaches remain largely symptomatic for IBS, are frequently compromised by non-response and disease recurrence in IBD, and often prove ineffective in advanced CRC. These persistent clinical challenges underscore an urgent need for novel conceptual frameworks that transcend traditional single-organ pathology perspectives.

To address this gap, a clear conceptual distinction is necessary. It is crucial to distinguish the GM-ENS axis from the broader microbiota-gut-brain axis. While the latter involves central neural processing with significant time delays, the GM-ENS axis operates as a local, high-speed, and semi-autonomous reflex circuit. Embedded within the gut wall, the ENS integrates microbial signals in real-time to orchestrate motility, secretion, and blood flow independent of immediate brain input (Macpherson et al., 2023; Grundeken and El Aidy, 2025). This review specifically focuses on this peripheral hub, arguing that its dysregulation constitutes the proximal cause of many digestive disorders, whereas central mechanisms often play a modulatory, secondary role.

Building on these concepts, we now focus specifically on the GM-ENS axis. A growing body of evidence – ranging from germ-free animal models to human translational studies – has established that the local, bidirectional crosstalk between GM and the ENS is not merely a component of the broader gut-brain axis, but rather the proximal driver of pathophysiology in IBS, IBD, and CRC. The ENS is embedded within the gastrointestinal wall. Unlike a passive relay, the ENS actively performs three core functions: (i) signal transduction – converting microbial metabolites and immune signals into neuronal firing patterns; (ii) local circuit integration – processing these inputs through intrinsic reflex arcs to coordinate motility and secretion independent of the brain; and (iii) effector command – issuing precise outputs to smooth muscle, epithelium, and vasculature via specific neurotransmitters. This intricate dialogue between microbiota and ENS is primarily mediated by the synergistic action of three pillars: microbial metabolites (e.g., bile acids), neuroactive substances (e.g., GABA, 5-HT), and immune signals (e.g., cytokines). Metabolites can both directly activate neuronal receptors and induce long-term epigenetic remodeling of the ENS, while neurotransmitters enable rapid signaling to regulate motility and sensation, and immune pathways act as crucial signal transducers linking dysbiosis to neuroinflammation.

Dysregulation of this network is now recognized as a key driver across major digestive disorders. The microbiota, via the ENS, influences three core pathophysiological features: motility disorders, visceral hypersensitivity, and immune dysregulation. A critical unanswered question remains: what are the relative contributions and specific molecular mechanisms through which the GM-ENS axis influences these features across different diseases or disease subtypes? Critically, we argue that the answers to this question will reveal distinct, disease-specific pathophysiological signatures.

This review systematically synthesizes the molecular mechanisms of GM-ENS interactions in digestive diseases, focusing on metabolite-mediated signaling, neuroimmune networks, and neurohormonal integration. We delineate the distinct pathophysiological roles of this axis across major digestive disorders: in IBS, it acts as a “signal distorter” driving visceral hypersensitivity and dysmotility; in IBD, it becomes an “inflammation amplifier” perpetuating neuroimmune dysregulation and neuronal injury; and in CRC, it functions as a “tumor accomplice” remodeling the microenvironment to promote cancer progression. By integrating these mechanistic insights, we chart a course from mechanistic exploration toward clinical translation. Our core objectives are threefold: first, to identify combinatorial microbiome-based biomarkers for precise disease classification and treatment response prediction; second, to refine targeted microbial interventions, moving from broad approaches like fecal microbiota transplantation(FMT) toward precision strategies such as synthetic microbial consortia or phage therapy; and third, to pioneer novel therapeutics targeting the “microbiota-ENS axis,” offering new hope for treating intractable digestive disorders.

## 2 The role of the ENS in the digestive tract

### 2.1 Structure and function of the ENS in the digestive tract

The ENS is an extensive neural network embedded within the gastrointestinal wall. It comprises two main plexuses: the myenteric and submucosal plexuses (Mazzone and Farrugia, 2007). The myenteric plexus, situated between the longitudinal and circular muscle layers, governs intestinal motility. Its cholinergic neurons stimulate contraction via acetylcholine, while nitrergic neurons induce relaxation through nitric oxide. In contrast, the submucosal plexus regulates mucosal functions, including secretion, local blood flow, and ion transport, and maintains the epithelial barrier via neuropeptides like vasoactive intestinal peptide (VIP). The ENS contains a diverse array of motor, sensory, and interneurons that form intrinsic circuits and connect bidirectionally with the central nervous system to integrate digestive processes with overall physiology. Supporting these neurons, glial cells secrete neurotrophins and modulate neurotransmitter metabolism to sustain neuronal survival and synaptic plasticity.

### 2.2 Techniques for analyzing the ENS

Comprehensive assessment of the ENS requires integrating multiple technical approaches, each with distinct advantages and limitations. Tissue biopsy (e.g., full-thickness or submucosal biopsy) combined with immunohistochemistry enables direct, high-resolution visualization of ganglionic and nerve fiber morphology. However, it is invasive, prone to sampling error, and precludes dynamic *in vivo* monitoring. Imaging techniques, such as optical imaging, offer non-invasive, real-time recording of neuronal activity with high spatial resolution, yet they lack the temporal resolution needed to capture fast neural electrical signals (Boesmans et al., 2015). Gastrointestinal motility testing indirectly assesses ENS function by measuring parameters like contraction frequency and conduction velocity. While useful for overall functional assessment, it cannot precisely localize the site of neuropathy (Kornum et al., 2021). Molecular biology techniques, including gene sequencing and proteomics, provide high sensitivity and specificity for elucidating underlying mechanisms. Their drawbacks include technical complexity, high cost, and stringent experimental requirements. Neurophysiological examinations directly record ENS electrical activity, making them essential for functional evaluation. However, they typically require electrode implantation or complex apparatus, which may cause tissue damage and compromise experimental stability (Rubaiy, 2017). In summary, no single technique suffices; a complementary approach is necessary in both clinical and research settings. Future technological development should strive to balance spatial and temporal resolution, invasiveness, and throughput, enabling a more systematic understanding of ENS regulatory mechanisms in health and disease and ultimately supporting precision diagnosis and treatment.

### 2.3 The ENS and digestive system diseases

**Figure 1:**
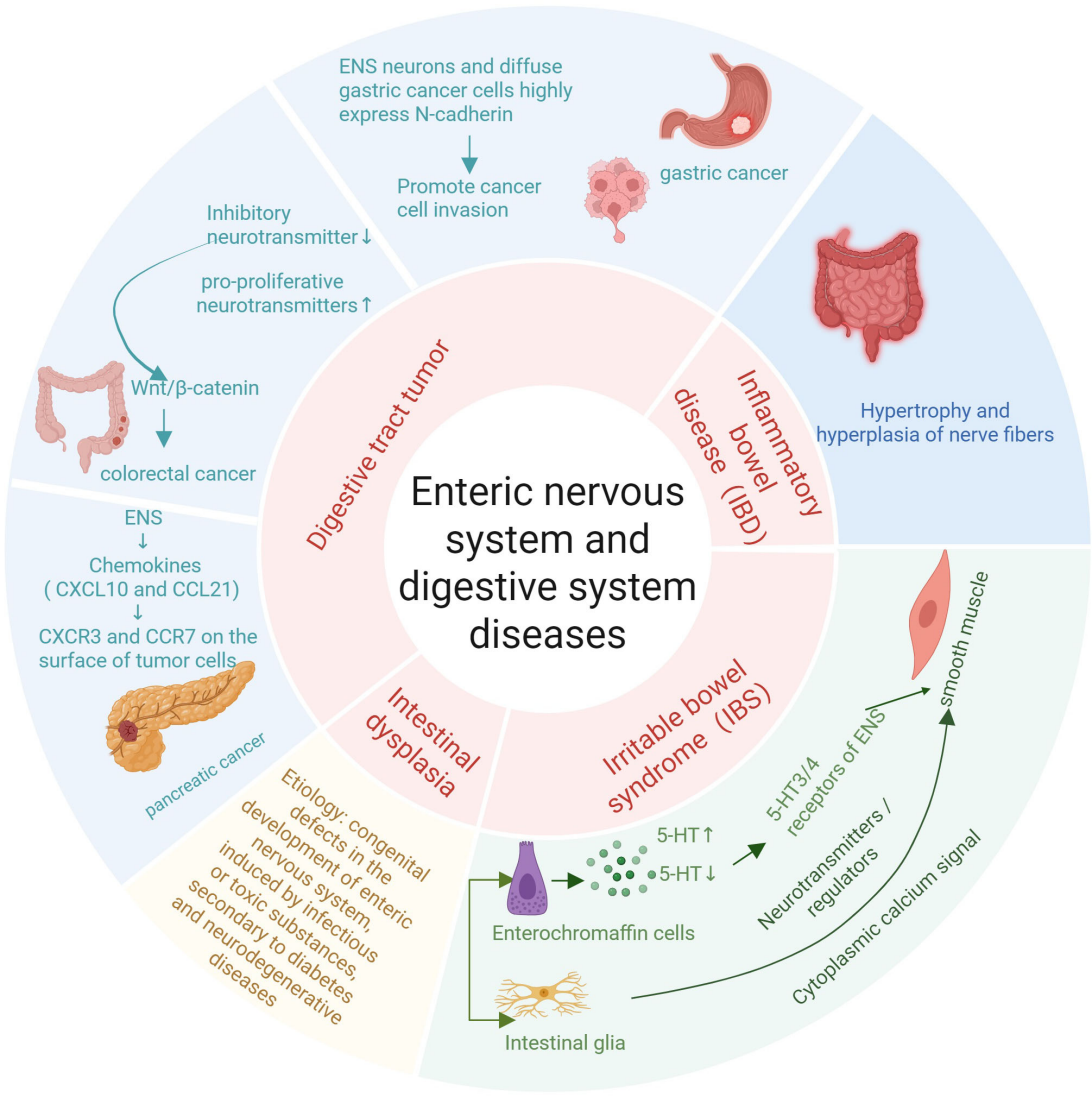
Diseases of the ENS and digestive system. Elucidating the relationships between the ENS and digestive health and disease, including abnormal gut development, irritable bowel syndrome (IBS), inflammatory bowel disease (IBD), and gastrointestinal (GI) tumors. (created with BioRender.com).

The ENS plays a pivotal role in digestive diseases, including IBS, IBD, and gastrointestinal tumors (**Figure 1**). These conditions involve not only intestinal dysfunction but also pathological changes in enteric neurons and glial cells.

#### 2.3.1 Abnormal intestinal development

ENS disorders arise from the loss, degeneration, or dysfunction of enteric neurons, with causes ranging from congenital developmental defects to acquired injuries (e.g., from infection or toxins) and secondary changes in systemic diseases like diabetes (Rao and Gershon, 2016). During development, enteric neural crest-derived progenitor cells must migrate, proliferate, and differentiate in a precisely timed manner to form a functional network within the gut wall. Failure of this process leads to aganglionic segments, manifesting as conditions such as esophageal atresia or Hirschsprung’s disease (HSCR) (Geng et al., 2022).

HSCR, affecting approximately 1 in 5,000 live births, is characterized by the absence of distal enteric ganglia, resulting in impaired meconium passage, intestinal obstruction, and severe constipation. Genetic studies have identified key genes involved in HSCR pathogenesis, including RET (Edery et al., 1994; Angrist et al., 1995), EDNRB (Puffenberger et al., 1994),SOX10 (Herbarth et al., 1998; Sham et al., 2001), PHOX2B (Garcia-Barceló et al., 2003), and the semagrin family (Luzón-Toro et al., 2013; Jiang et al., 2015).These genes regulate neural crest cell migration, proliferation, and differentiation through highly conserved signaling pathways; their disruption ultimately leads to congenital intestinal obstruction (Heuckeroth, 2018). More recent studies have identified additional candidate genes and genetic variants contributing to HSCR susceptibility (Tilghman et al., 2019; Mederer et al., 2020; Kuil et al., 2021).

Beyond HSCR, ENS abnormalities underlie several other gastrointestinal disorders. Achalasia is characterized by loss of esophageal peristalsis and impaired relaxation of the lower esophageal sphincter (LES). Studies indicate that a marked reduction in inhibitory neurons within the distal esophageal ENS is a key pathological driver (Boeckxstaens, 2016; Rieder et al., 2020) Gastroparesis, defined by delayed gastric emptying in the absence of mechanical obstruction, is associated with significant loss of interstitial cells of Cajal (ICC) and neuronal fibers in the gastric circular muscle layer, alongside increased immune infiltration of the myenteric plexus (Grover et al., 2011). These findings collectively highlight that ENS abnormalities are central to the pathogenesis of diverse enteric neuropathies.

#### 2.3.2 IBS

IBS is a functional bowel disorder characterized by abdominal pain, bloating, and altered bowel habits, with pathogenesis linked to brain-gut axis dysfunction, visceral hypersensitivity, and low-grade inflammation—all closely tied to ENS abnormalities. IBS is subtyped into diarrhea-predominant (IBS-D), constipation-predominant (IBS-C), and mixed (IBS-M). These distinct phenotypes reflect underlying differences in ENS-mediated motility. In IBS-D, increased 5-HT secretion from enterochromaffin cells(ECCs) activates ENS 5-HT3/4 receptors, driving colonic hypermotility and accelerated transit. Conversely, IBS-C patients exhibit reduced 5-HT levels, associated with delayed colonic transit and gastric emptying (Pastras et al., 2024). This dysregulation manifests as distinct motility patterns: IBS-D features high-amplitude propulsive contractions, whereas IBS-C shows a predominance of segmental contractions, directly influencing stool consistency and defecation frequency. Notably, stool consistency correlates positively with colonic transit time, underscoring the functional impact of ENS-mediated motility disturbances in IBS pathophysiology.

ENS dysfunction interacts with immune and endocrine signals to exacerbate IBS symptoms. Mast cell-derived histamine activates neuronal TRPV1 channels, inducing visceral hypersensitivity, while eosinophils enhance ENS synaptic plasticity via nerve growth factor (NGF) (Tozlu et al., 2021; Hasler et al., 2022; Chen et al., 2023). Short-chain fatty acids(SCFAs) and peptide YY (PYY) regulate intestinal sensitivity (El-Salhy et al., 2020) by inhibiting colonic peristalsis and increasing 5-HT synthesis, respectively. Clinically, abdominal distension correlates positively with orofecal transit time, whereas pain and flatulence do not, suggesting that sensory and motor pathways within the ENS are independently regulated.

Enteric glial cells(EGCs), key components of the ENS, are closely implicated in IBS-D pathophysiology. EGCs regulate intestinal motility through at least two mechanisms: activation of intracellular Ca^2+^ signaling, and expression of neurotransmitter receptors that modulate smooth muscle contractility in response to ENS signals. Under inflammatory conditions, EGC proliferation increases. Animal studies confirm that abnormal proliferation and activation of EGCs in the colonic myenteric plexus of IBS-D rats directly affect colonic contractility; importantly, inhibiting EGC activation ameliorates this motor dysfunction. Mechanistically, this involves bidirectional communication between enteric neurons and EGCs. EGCs surrounding neurons monitor neural activity via neurotransmitter receptors. Specifically, purines released from enteric neurons act on EGC P2Y1 receptors, triggering downstream pathways that regulate colonic contractility. This neuron–EGC signaling axis represents a key mechanism by which EGCs contribute to the pathogenesis of IBS-D (Li et al., 2023).

Together, these findings illustrate that in IBS, the ENS and its associated regulatory network function as a “signal distorter” —amplifying or dampening neural signals to drive visceral hypersensitivity and dysmotility. We propose that this distortion manifests as a subtype-dependent shift in the excitatory/inhibitory balance: a tipping toward cholinergic excitation in IBS-D (leading to hypermotility), and a dominance of nitrergic inhibition in IBS-C (causing hypomotility). The mechanistic framework predicts that restoring this ratio, rather than absolute levels of any single neurotransmitter, should be the therapeutic goal, a strategy we will detail in Section 5.

However, it is important to note that most evidence linking microbiota-derived metabolites to IBS symptom generation comes from animal models or cross-sectional human studies. Whether the observed shifts in excitatory/inhibitory balance are causal or epiphenomenal remains unclear. Moreover, the relative contribution of microbial versus host genetic factors to this imbalance has not been systematically addressed, and longitudinal studies tracking the same individuals across symptom flares and remissions are urgently needed to establish causality.

#### 2.3.3 IBD

IBD, encompassing Crohn’s disease (CD) and ulcerative colitis (UC), is characterized by chronic gastrointestinal inflammation with prominent ENS involvement, including neuronal loss, nerve fiber damage, plexitis (e.g., myenteric plexitis), and EGC proliferation. While IBD pathogenesis traditionally emphasizes genetic susceptibility interacting with environmental, microbial, and immune factors, emerging evidence points to significant ENS remodeling. Inflamed intestinal segments from IBD patients exhibit nerve fiber hypertrophy and hyperplasia in the mucosa, submucosa, and myenteric plexus, accompanied by inflammatory infiltration, submucosal structural defects, fiber retraction, and neuroma-like lesions. These observations raise the question of whether ENS structural changes are merely a consequence of inflammation or actively contribute to IBD pathogenesis. Functional consequences of this remodeling are substantial. ENS abnormalities disrupt intestinal motility, secretion, and sensation, directly exacerbating clinical symptoms such as abdominal pain and dysmotility. Moreover, these peripheral neural changes may propagate systemically via neural pathways, contributing to extraintestinal manifestations including movement disorders and altered pain perception. Conversely, IBD-related inflammation reciprocally affects ENS structure and function, reducing acetylcholine release while increasing proinflammatory neurotransmitters like 5-HT, thereby perpetuating a vicious cycle of neuroinflammation and intestinal dysfunction (Suman, 2024).

The mechanistic role of the ENS in IBD remains underexplored. However, recent evidence shows that IBD risk genes and cytokine signaling-related genes are enriched in the ENS, implicating a direct involvement of the ENS in IBD pathogenesis (Drokhlyansky et al., 2020).

EGCs may also play a critical role in IBD. Under homeostatic conditions, EGCs maintain intestinal health by secreting neurotrophic factors, preserving epithelial barrier integrity (via tight junction regulation), and modulating immune cell activity (e.g., mast cells, macrophages). In IBD, however, proinflammatory cytokines activate EGCs, transforming them into reactive glial cells that release nitric oxide (NO), reactive oxygen species (ROS), and inflammatory mediators, leading to persistent intestinal gliosis. Notably, overproliferating EGCs may undergo oxidative DNA damage and telomere shortening, driving cellular senescence. These senescent EGCs acquire a senescence-associated secretory phenotype (SASP), releasing proinflammatory factors such as IL-1 and matrix metalloproteinases (MMPs). This SASP response contributes critically to IBD onset, fuels chronic inflammation, and promotes disease recurrence (Kumar et al., 2023).

Recent studies reveal that intestinal sensory neurons (PSNs) directly sense immune signals (such as type 2 cytokines during parasitic infection) by expressing IL-4/IL-13 receptors. During parasitic infection, type 2 cytokines activate these neurons, upregulating the neuropeptides NMU and CGRPβ. NMU activates ILC2 cells to secrete IL-5/IL-13, recruiting eosinophils for pathogen elimination. Concurrently, CGRPβ exerts dual functions: it inhibits excessive ILC2 proliferation to prevent tissue damage, while synergizing with IL-4 to induce arginase-1 (Arg1) expression in muscularis macrophages, thereby promoting parasite clearance and tissue repair. This bidirectional neuro-immune axis precisely regulates intestinal inflammation. Its dysregulation leads to immune defense defects and chronic inflammation, as observed in IBD (Barilla et al., 2025).

Taken together, the evidence positions the ENS in IBD as an ‘inflammation amplifier’. We propose a two-stage loop: Stage 1 (Initiation): Dysbiosis-induced epithelial breach activates ENS sensory neurons. Stage 2 (Amplification): Activated ENS neurons release substance P and CGRP, which stimulate mast cells and macrophages to produce TNF-α and IL-1β. These cytokines, in turn, further activate ENS neurons and disrupt barrier function, creating a positive feedback loop. This framework identifies the ENS itself as a potential therapeutic target to ‘break the cycle’. Strategies to disrupt this amplification loop are discussed in Section 5.

Nevertheless, several key questions remain unresolved. First, it is unclear whether ENS activation is a driver or a consequence of intestinal inflammation – animal studies suggest bidirectional causality, but human data are lacking (van Baarle et al., 2023; Suman, 2024). Second, most mechanistic insights derive from acute colitis models, whereas human IBD is chronic and relapsing; whether the same amplification loop operates in chronic settings requires further investigation (Zeeff et al., 2016).

#### 2.3.4 Tumors of the digestive tract

The ENS is increasingly recognized as a modulator of tumorigenesis and progression in gastrointestinal cancers, including pancreatic, colorectal, and gastric cancer. Aberrant ENS signaling may promote abnormal cell proliferation and create a permissive microenvironment for malignancy.

Pancreatic cancer, known for the highest incidence of neural invasion among all cancers, provides the most striking example of ENS involvement. Schwann cells, the principal glial cells of the peripheral nervous system, undergo dynamic activation in response to pancreatic cancer. They proliferate and migrate toward tumor cells, initiating nerve–tumor cell interactions that facilitate neural invasion. Beyond direct contact, Schwann cells also crosstalk with tumor-associated macrophages, further promoting this process (Jiang et al., 2025). ENS neurons secrete chemokines such as CXCL10 and CCL21; pancreatic cancer cells, which highly express the corresponding receptors (CXCR3 and CCR7), are chemotactically attracted to ENS neurons, promoting local spread and metastasis (Hirth et al., 2020). Conversely, pancreatic cancer cells release neurotrophic factors like NGF, which stimulate ENS neuronal proliferation and activity, creating a vicious cycle that exacerbates invasion. ENS activation also triggers neural remodeling. Exposure to pancreatic cancer cell supernatant induces marked neurite outgrowth, increased neurite density, and perinuclear hypertrophy in myenteric plexus and dorsal root ganglion neurons (Demir et al., 2010). This pathological remodeling not only aggravates the proinflammatory and immunosuppressive state of the tumor microenvironment but may also directly contribute to cancer-related pain through release of pain mediators.

CRC is the second leading cause of cancer-related death worldwide, and emerging evidence implicates the ENS in its pathogenesis. For instance, expression of netrin-1—a tumor suppressor protein synthesized by intestinal neurons during gastrointestinal organogenesis—is decreased in CRC patients. CRC invasion disrupts normal intestinal architecture and establishes a tumor microenvironment (TME) that induces ENS atrophy. This neurodegeneration creates a vicious cycle of cancer progression, characterized by an imbalance in ENS-derived neurotransmitters. Inhibitory neuropeptides such as VIP and neuropeptide Y (NPY) are downregulated, weakening both motility regulation and anti-inflammatory effects. Conversely, pro-proliferative neurotransmitters like acetylcholine (ACh) are upregulated within the TME. Elevated ACh promotes cancer cell proliferation and migration through multiple signaling pathways. It activates the Wnt/β-catenin pathway, a key driver of CRC progression (Konishi et al., 2019). Additionally, ACh binding to its receptors induces matrix metalloproteinase 7 (MMP-7) release (Xie et al., 2009), which in turn activates EGF/EGFR signaling and downstream cascades including PI3K/PKC/NF-κB and Ras/Raf/MEK/MAPK/ERK, ultimately enhancing cell proliferation (Godlewski and Kmiec, 2020).

Beyond enteric neurons, EGCs also contribute to the CRC TME. Upon stimulation by tumor-derived ligands, EGCs can transdifferentiate into a pro-cancer phenotype, enhancing the tumorigenic potential of cancer stem cells (CSCs) and thereby driving tumor initiation. Additionally, S100B protein expression is significantly elevated in both serum and tumor tissues of CRC patients. Elevated S100B shapes a malignant microenvironment through multiple mechanisms: inducing proinflammatory cytokine release, stimulating VEGF-mediated tumor angiogenesis, and upregulating anti-apoptotic proteins that increase tumor cell chemoresistance (Seguella et al., 2020).

The ENS also plays a key role in gastric cancer (GC) progression, through both physical adhesion and signaling interactions between tumor cells and enteric neurons. Studies demonstrate that diffuse-type GC cells exhibit stronger adhesion to ENS neurons than intestinal-type GC cells, potentially explaining the higher invasiveness and perineural invasion tendency of diffuse-type GC. Using a coculture model of gastric ENS and GC cells, researchers found that GC cells preferentially adhered to neuronal surfaces, whereas adhesion was significantly reduced in neuron-depleted cultures, confirming that neurons are essential for this process. Mechanistically, N-cadherin mediates this adhesion. N-cadherin is highly expressed on both ENS neurons and diffuse-type GC cells, and blocking it with a specific antibody significantly inhibits tumor–neuron adhesion. These findings suggest that N-cadherin promotes diffuse GC spread along neural pathways by facilitating heterotypic adhesion between tumor cells and neurons. Beyond adhesion, neurons within the ENS microenvironment may further promote GC progression by releasing neurotrophic factors or inflammatory mediators (e.g., acetylcholine), potentially activating cancer stem cell phenotypes. Collectively, the ENS contributes to GC invasion through both N-cadherin-dependent physical adhesion and cholinergic signaling pathways (Girot et al., 2022).

In summary, the ENS assumes the role of a “tumor accomplice” across multiple gastrointestinal malignancies. In CRC, this occurs through a bidirectional, self-reinforcing loop: tumor-derived factors remodel ENS neurotransmitter balance and glial phenotype, while the remodeled ENS promotes cancer stemness, invasion, and chemoresistance via Wnt/β-catenin and MMP-7/EGFR pathways. In gastric cancer, the ENS acts through N-cadherin-mediated physical adhesion and cholinergic signaling. In pancreatic cancer, it contributes via chemokine-guided neural attraction (CXCL10–CXCR3, CCL21–CCR7) and Schwann cell-driven neural remodeling. These distinct mechanisms illustrate that the “accomplice” role is molecularly tailored to each tumor type. The therapeutic implications of targeting this neural–cancer crosstalk are explored in Section 5.

Despite growing evidence, causal relationships remain difficult to establish in human CRC, where tumor–ENS interactions are confounded by treatment history, tumor heterogeneity, and intraindividual microbial variability. Most functional data derive from cell lines or xenograft models, which may not recapitulate the complex tumor microenvironment in patients. Whether targeting ENS-derived signals will yield therapeutic benefits without disrupting normal gut physiology remains an open question.

## 3 Interaction between the gut microecology and ENS

Although early studies predominantly focused on unidirectional influences between the GM and the ENS, recent evidence indicates that both are in fact embedded within a complex bidirectional signaling circuit, engaging in real-time reciprocal feedback.

### 3.1 Composition and function of the GM

The GM is an organic dynamic balance state (Roy and Dhaneshwar, 2023), which is composed of many different microorganisms and their living environments, such as intestinal epithelial cells and the intestinal mucosal immune system (Yan and Wu, 2023). The GM is a complex community composed of bacteria, fungi and viruses, and its core functions include metabolic regulation, immune regulation and barrier protection. The intestinal microbiota ferments dietary fiber to produce SCFAs, such as acetic acid, propionic acid, and butyric acid. These metabolites not only provide energy for intestinal epithelial cells but also participate in regulating immune responses and maintaining the integrity of the intestinal barrier. In addition, intestinal microbes are essential for host metabolism, blood coagulation, and other physiological functions through the synthesis of vitamins, such as B and K (Jandhyala et al., 2015). Under physiological conditions, the human body and GM exist in an interdependent “dynamic balance”. The two interact through complex metabolism and signals to jointly build the body’s health defense line. Once this delicate balance is disrupted, disordered GM triggers a chain reaction, which is a hidden danger for the occurrence and development of a variety of diseases.

### 3.2 Influence of the GM on the ENS

Traditionally, the GM is thought to influence the ENS through three major classes of signaling molecules – microbial metabolites (e.g., SCFAs), neuroactive substances (e.g., GABA, 5−HT), and immune mediators – acting over minutes to hours to maintain gut homeostasis. However, two recent breakthroughs have dramatically expanded our view of the speed and dimensionality of microbiota–ENS communication: millisecond−scale synaptic transmission via neuropod cells, and bacterial galvanotaxis driven by endogenous bioelectric fields. Together, these mechanisms form a multi−timescale, multimodal regulatory hierarchy, enabling the ENS to integrate signals ranging from fast reflexes to long−term remodeling.

Many experiments have revealed the close relationship between the GM and the host gut. Taking antibiotic (ABX)-treated mice as an example, ABX-treated mice present a series of structural and functional changes in the gastrointestinal tract. The small intestine is significantly longer, the intestinal transit time is significantly slower, and the intestinal permeability also tends to increase. At the neuroanatomical level, ABX treatment results in massive loss of enteric neurons in the submucosal and myenteric plexuses of the ileum and proximal colon, but only in the myenteric plexus of the ileum is a reduction in the number of EGCs observed (Vicentini et al., 2021; Kato et al., 2024). The possible mechanism of this effect was further investigated.

#### 3.2.1 GM -derived metabolites

**Figure 2:**
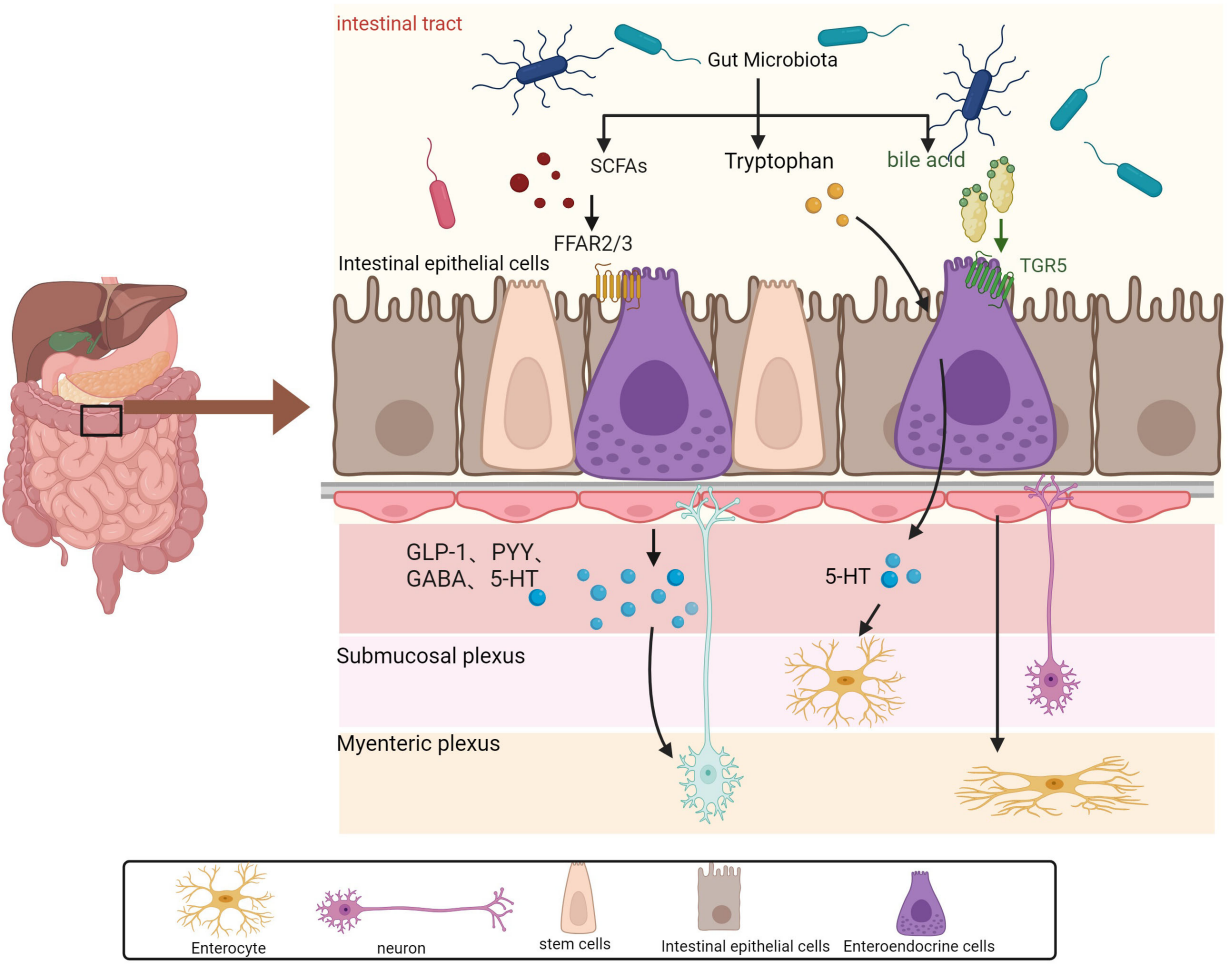
Effects of gut microbiota-derived metabolites on the ENS. This figure illustrates that the gut microbiota communicates with the ENS through its metabolites, regulating gut function and health. For example, SCFAs influence the neural activity of the ENS through the production of GLP-1, PYY, GABA and 5-HT via the free fatty acid receptors FFAR2/FFAR3 on enteroendocrine cells, and secondary bile acids regulate neural signaling via the bile acid receptor (TGR5, etc.). (created with BioRender.com).

The GM produce metabolites such as SCFAs and bile acids that exert profound effects on the ENS through multiple mechanisms (**Figure 2**). These substances can act directly on ENS neurons or influence the CNS via circulatory or neuroendocrine pathways (Pan et al., 2022).

SCFAs, particularly butyrate, regulate ENS function through at least three distinct mechanisms: epigenetic modulation, receptor-mediated signaling, and metabolic support. At the epigenetic level, SCFAs inhibit histone deacetylases (HDACs), with butyrate exhibiting the most potent activity (Cleophas et al., 2016). HDAC inhibition increases histone acetylation, opening chromatin structure and regulating transcriptional programs of genes involved in neuronal survival, differentiation, and synaptic plasticity (e.g., brain-derived neurotrophic factor, BDNF). This mechanism is critical for ENS development and adaptive remodeling. At the receptor level, SCFAs bind to G protein-coupled receptors, including free fatty acid receptor 2 (FFAR2) and free fatty acid receptor 3(FFAR3), expressed on intestinal endocrine cells (Kuwahara et al., 2018). This triggers intracellular calcium mobilization and cAMP alterations, stimulating the release of neuroactive peptides and neurotransmitters such as glucagon-like peptide-1 (GLP-1), peptide YY (PYY), GABA, and 5-HT (Panaro et al., 2014). These mediators directly modulate intestinal motility and sensation. Metabolically, SCFAs serve as preferred substrates for mitochondrial β-oxidation in enteric neurons and glia, efficiently generating ATP to support high-energy processes including action potential conduction, neurotransmitter recycling, and synaptic transmission (Donohoe et al., 2011). This metabolic support is particularly important for maintaining ENS functional stability under stress conditions.

It is critical to note that most of this evidence comes from *in vitro* studies or rodent models. While butyrate’s HDAC inhibition is robust in cell lines, its relevance in the intact human ENS under physiological conditions is less certain. Moreover, conflicting reports exist on whether butyrate excites or inhibits enteric neuron activity – likely due to differences in concentration, exposure time, and presence of inflammation. A major unanswered question is: under normal physiology, does butyrate primarily act via HDAC inhibition (epigenetic, slow, long−lasting) or via GPCR signaling (FFAR2/3, fast, reversible) to regulate ENS function? Resolving this will require tools to distinguish these pathways *in vivo* – for example, conditional knockout of HDAC or FFARs in ENS neurons combined with real−time calcium imaging during colonoscopy in live animals. Such experiments would move the field from correlation to mechanism. Collectively, this multi-layered regulatory network ensures ENS development and homeostasis; its dysregulation is implicated in gastrointestinal motility disorders, visceral hypersensitivity, and neuroinflammation.

The GM convert primary bile acids (synthesized by the liver) into secondary bile acids via enzymatic modification. Bile acids influence ENS signaling through two principal pathways. First, they activate the G protein-coupled receptor TGR5, which is widely expressed on enteric neurons and glial cells. TGR5 activation triggers the cAMP-PKA signaling cascade, modulating neuronal excitability, synaptic plasticity, and neurotransmitter release (e.g., acetylcholine, VIP) (Poole et al., 2010). This mechanism stimulates intestinal smooth muscle contraction and accelerates transit. Animal studies confirm that the secondary bile acid lithocholic acid (LCA) enhances colonic motility via TGR5, whereas TGR5 knockout mice exhibit impaired motility. Second, bile acids activate the nuclear receptor farnesoid X receptor (FXR), expressed in both enteric neurons and glial cells (Mertens et al., 2017). Although FXR’s neuroprotective role in the CNS is established, its precise function within the ENS requires further investigation.

#### 3.2.2 GM-derived neurochemicals

The GM modulates ENS function by influencing the synthesis and metabolism of key neurotransmitters, including 5-HT, GABA, and dopamine (Asano et al., 2012; Barrett et al., 2012).

Approximately 90% of the body’s 5-HT is produced by ECCs via tryptophan hydroxylase 1 (TPH1), with the remainder derived from neuronal TPH2 in the myenteric plexus (Strege et al., 2017). 5-HT is a critical regulator of intestinal motility, enhancing peristalsis. The GM influence 5-HT signaling through multiple mechanisms. First, SCFAs promote TPH1 transcription in human ECCs and activate 5-HT4 receptor-expressing neurons in the submucosal and myenteric plexuses (Yano et al., 2015). Second, intestinal microorganisms such as Bifidobacterium and Lactobacillus metabolize tryptophan to produce indole derivatives (e.g., indolepropionic acid), which modulate host tryptophan metabolism and thereby affect 5-HT synthesis. 5-HT exerts its effects on ENS function by activating multiple receptors, including 5-HT3 and 5-HT4. For example, 5-HT activates ENS neurons to promote myenteric contraction and accelerate intestinal peristalsis. Dysbiosis-associated alterations in 5-HT levels may contribute to constipation or diarrhea.

The GM can also produce GABA from glutamate and glutamine, directly influencing host neural signaling. GABA exerts dual excitatory and inhibitory effects on the ENS through distinct receptor subtypes. Activation of GABA-A receptors—ligand-gated chloride channels—induces chloride influx, hyperpolarizing enteric neuron membranes, reducing neuronal excitability, and inhibiting action potential generation. Conversely, GABA-C receptor activation may produce excitatory effects via sustained depolarizing currents sufficient to activate voltage-gated calcium channels. Functionally, GABA regulates intestinal motility through both direct and indirect mechanisms. GABAergic neurons form presynaptic connections with motor neurons innervating intestinal smooth muscle. By releasing GABA, they inhibit neurotransmitter release from motor neurons: inhibition of cholinergic neurons attenuates contraction, whereas inhibition of nitrergic neurons enhances it. This indirect, network-level regulation exemplifies the integrative capacity of the ENS as an independent nervous system, enabling flexible modulation of intestinal motility and secretion.

While these mechanisms are well-supported by *in vitro* and animal studies, their translational relevance to human digestive diseases requires caution. For example, the contribution of microbiota-derived GABA to luminal GABA pools versus host-derived GABA remains unclear, as does whether bacterial GABA can cross the epithelial barrier to reach ENS neurons. Furthermore, most studies have focused on a handful of bacterial species (e.g., Lactobacillus, Bifidobacterium); whether the diverse human gut microbiome produces neuroactive compounds at physiologically relevant concentrations is largely unknown. Future studies should combine gnotobiotic models with human validation cohorts to establish causality. Collectively, these mechanisms illustrate that the ENS–microbiota relationship is not a unidirectional causal chain but a complex, bidirectional, and self-amplifying network.

#### 3.2.3 Critical periods of development: the programming role of microbiota on the ENS and its temporal specificity

The microbiota can regulate the intestinal environment, and studies have shown that the microbiota is required for the postnatal development of mucosal EGCs (Kabouridis et al., 2015). The GM is critical for the maturation and maintenance of the ENS during the early postnatal period. Significant abnormalities in the myenteric plexus of the jejunum and ileum, characterized by decreased nerve density, a decreased number of neurons per ganglion, and an increased proportion of nitrogen-containing neurons in the myenteric plexus, were detected in germ-free mice (Collins et al., 2014). These findings demonstrate that ENS development is severely impaired in the absence of microbiota. Further studies, which disrupted the GM by administering vancomycin during the critical developmental period after weaning, profoundly revealed the role of microbiota in shaping the ENS. Research confirms that this ‘germ-free’ state induces specific structural and functional deficits in the ENS: not only does it significantly reduce the number of key intrinsic sensory neuronal subpopulations, such as cholinergic neurons, disrupting the coordinated network of intestinal motility, but it also directly leads to functional consequences, including a decrease in the propagation velocity of the colonic migratory motor complex. Crucially, this work demonstrates strict timing specificity in microbiota programming of the ENS. The same antibiotics administered post-weaning rather than during neonatal stages induced diametrically opposed, even contradictory, motor phenotypes and neurochemical alterations, underscoring early life as an irreversible programming window. At the mechanistic level, the study indicates that the microbiota likely executes this programming via its metabolites. This is suggested by significantly elevated colonic tryptophan levels in treated mice, indicating a metabolic pathway shift. Concurrently, alterations in microbiota composition—such as the expansion of Akkermansia and Lactobacillus species—imply that microbial metabolites, including SCFAs, may play a crucial role as neurotrophic factors in regulating neuronal excitability and ENS maturation (Hung et al., 2020). However, as these findings are primarily based on animal models, whether identical developmental windows and microbial signals exist in the human gut, and how these interact with the host’s genetic background, remain key questions for future research to explore.

#### 3.2.4 GM indirectly affects the ENS by transmitting immune signals

**Figure 3:**
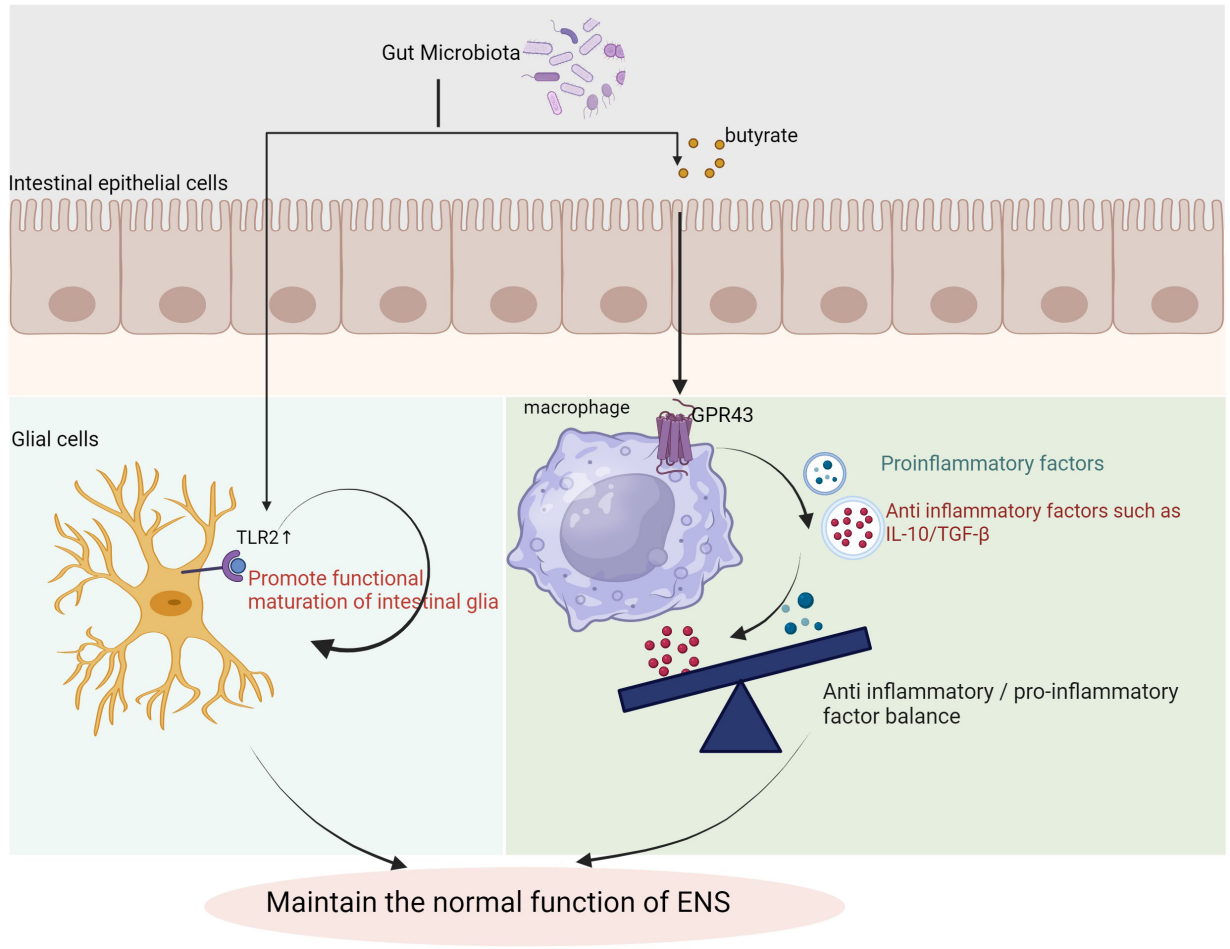
Gut microbiota affects the ENS by transmitting immune signals. This figure illustrates that the gut microbiota profoundly influences the structure and function of the ENS by activating the gut immune system and producing and releasing immune signaling molecules. The gut microbiota not only plays an important role in regulating ENS development and functional homeostasis through the TLR signaling pathway but also activates various intracellular signaling pathways by binding to the GPR43 receptor (FFAR2 receptor), which affects the balance of proinflammatory and anti-inflammatory factors and thus maintains the normal function of ENS neurons. (created with BioRender.com).

The ENS is anatomically and functionally integrated with gut-associated lymphoid tissue, positioning immune cells not only as defenders against pathogens but also as key signal transducers within the microbiota–ENS axis. Microbial signals can activate or polarize resident immune cells, prompting release of soluble factors that directly modulate the survival, excitability, and neurotransmitter phenotype of adjacent enteric neurons and glia (**Figure 3**).

Butyrate, a principal short-chain fatty acid derived from microbial fermentation, exemplifies this pathway. By activating the G protein-coupled receptor GPR43 on intestinal macrophages, butyrate inhibits NF-κB and modulates MAPK signaling, driving macrophages toward an anti-inflammatory phenotype and promoting secretion of IL-10 and TGF-β (Kim et al., 2013). These mediators protect ENS neurons from inflammatory assault and maintain a supportive microenvironment for neural function (Nogal et al., 2021).

Conversely, in IBD, pro-inflammatory cytokines such as TNF-α and IL-1β dominate. Activating their receptors on ENS neurons triggers p38 MAPK and NF-κB pathways, leading to altered ion channel function, abnormal excitability, and eventually neuronal apoptosis. This pro-inflammatory milieu disrupts the balance maintained by microbiota-driven anti-inflammatory signals (e.g., IL-10), constituting a core pathological mechanism by which dysbiosis compromises ENS integrity.

Microbiota also directly program ENS development and homeostasis via toll-like receptors (TLRs). TLR2, expressed on enteric neurons, glia, and smooth muscle cells, is essential for ENS integrity. TLR2-deficient mice exhibit reduced nitrergic inhibitory neurons and acetylcholinesterase-positive fibers, impaired motility, decreased chloride secretion, and diminished glial markers (Nogal et al., 2021). Both probiotics and pathogens upregulate TLR2 on EGCs, suggesting that microbiota–TLR2 signaling promotes EGC functional maturation (Turco et al., 2014). Notably, glial cell line-derived neurotrophic factor (GDNF) is downregulated in TLR2-deficient mice, and exogenous GDNF rescues the ENS defects, implicating microbiota in regulating mesenchymal-derived neurotrophic factors.

Beyond classical metabolite- and immune-mediated pathways, recent studies reveal a more integrated microbiota–ENS dialogue. Using systems that simultaneously manipulate neuronal activity and microbial input, researchers have shown that Thomasclavella ramosa not only directly regulates enteric neuron excitability but also remodels downstream immune and barrier responses programmed by specific neuronal activities (Naim et al., 2025). This suggests that microbiota form a dynamic “regulatory layer” that integrates with real-time neuronal commands at immune and epithelial interfaces to fine-tune physiological output—a higher-order tripartite dialogue between microbes, neurons, and immune cells.

A major unresolved question is the extent to which immune-mediated microbiota–ENS signaling operates independently of direct neuronal sensing of microbial metabolites. Most studies have examined these pathways in isolation, but *in vivo* they likely act in parallel and redundantly. Thus, while immune signals are critical bidirectional transducers within the microbiota–ENS axis, dissecting their unique versus shared contributions with direct metabolite sensing represents a key priority for future research.

#### 3.2.5 Emerging paradigms: neuropod cells and bioelectric signaling

Beyond the classical triad of metabolites, neuroactive substances, and immune mediators, recent breakthroughs have uncovered additional, faster modes of microbiota-ENS communication.

Neuropod cells are specialized enteroendocrine cells that form direct, glutamatergic synapses with vagal and enteric neurons. This enables millisecond-scale signaling, in contrast to the minute-scale delays of hormonal routes (Kaelberer et al., 2018; Buchanan et al., 2022). Critically, these cells also sense gut microbes: colonic PYY^+^ neuropod cells express Toll-like receptor 5 (TLR5) that detects bacterial flagellin, triggering vagal activation via neuropeptide Y receptor type 2(NPY2R) to regulate feeding independently of overt immune responses (Liu et al., 2025b). This “neurobiotic sensing” paradigm reveals ultrafast microbiota-host communication, potentially explaining rapid symptom onset in disorders like IBS. However, while a direct gut-microbe–vagus–brain pathway is established, how neuropod cells directly engage the ENS – as opposed to central vagal circuits – to regulate local gut functions (motility, secretion, barrier integrity) remains an open question.

Beyond chemical signals, recent studies have explored bioelectricity as a potential communication modality. The intestinal epithelium generates endogenous electric fields that can guide bacterial swimming and localization – a phenomenon termed galvanotaxis (Sun et al., 2024). Concurrently, gut bacteria possess membrane potential dynamics and can perform long-range electrical communication via ion channels. These findings raise the possibility of bidirectional electrical crosstalk between the microbiota and the ENS. However, direct evidence for such electrical dialogue *in vivo* remains lacking, and whether endogenous bioelectric activities are sufficient to modulate ENS function is a critical open question.

These emerging modalities do not replace the classical triad of metabolites, neurotransmitters, and immune mediators, but rather add faster (millisecond-scale) and spatially organized layers of communication. Neuropod cells enable direct lumen-to-neuron synaptic transmission, bypassing the minute-long delays of hormonal routes, while bioelectric fields may provide a long-range, continuous guidance cue for bacterial positioning. Collectively, these findings suggest a multi-timescale hierarchical control system: rapid (ms–s) electrical/neuropod signaling for immediate reflexes, intermediate (min–h) metabolite/neurotransmitter action for homeostatic tuning, and slow (h–d) immune/plasticity changes for long-term remodeling. Future studies should investigate how these layers interact and whether their disruption contributes differentially to IBS (fast signaling distortions) versus IBD (slow immune amplification).

### 3.3 Effects of the ENS on GM

#### 3.3.1 The ENS can reshape the gut microbiome

The ENS actively shapes the gut microenvironment to influence microbial growth and metabolism through release of neurotransmitters such as acetylcholine, VIP, and monoamines.

5-HT, abundant in the gut, not only regulates intestinal peristalsis and secretion but also alters the physical and chemical environment, thereby affecting microbial niche space and nutrient availability. VIP, another key ENS neurotransmitter, acts on intestinal epithelial cells to regulate epithelial fucosylation. The resulting fucosylated glycans serve as adhesion sites and nutrient sources for commensal microbes, influencing their colonization and growth. Dysregulated VIP signaling leads to depletion of beneficial bacteria and outgrowth of harmful species (Lei et al., 2022). Recent studies using targeted activation of specific ENS neuron populations in mice reveal that only choline acetyltransferase (ChAT)-expressing neurons—not tyrosine hydroxylase (TH)-expressing neurons—increase colonic contractility and induce diarrhea-like fluid secretion upon activation. These findings demonstrate that distinct peripheral neuron subsets regulate the gut microbiome independently of brain signals, highlighting the ENS’s autonomous role in shaping microbial communities (Griffiths et al., 2024).

Stress directly impairs ENS function and disrupts intestinal epithelial tight junctions, increasing gut permeability (“leaky gut”). This barrier dysfunction promotes dysbiosis, characterized by overgrowth of opportunistic pathogens and altered microbial metabolite profiles (Di Vincenzo et al., 2024).

ENS signals also regulate intestinal epithelial cells to secrete antimicrobial peptides and modulate mucus production. These defense elements directly inhibit or kill pathogenic bacteria while maintaining a protective barrier that influences microbial distribution and survival (Dowling et al., 2022).

#### 3.3.2 Effects on GM through neuroimmune modulation

Intestinal immunity is an important factor in maintaining the stability of the GM. The ENS can indirectly shape the GM by transmitting immune signals. In turn, the ENS can regulate the activities of immune cells such as macrophages, dendritic cells and T lymphocytes by releasing neurotransmitters and neuropeptides, such as phagocytosis by macrophages and cytokine secretion, and guide the distribution and migration of immune cells in the intestine so that they can quickly reach the site of infection or inflammation and subsequently affect the local immune status of the intestine (Chandrasekharan et al., 2013). Second, the ENS can affect the type of immune response of the immune system to intestinal microorganisms, maintain the balance of Th1/Th2, and play a role in the development and maturation of the intestinal immune barrier, thereby maintaining the stability of the GM in many ways.

## 4 Effects of interactions between the GM and the ENS on the digestive system

### 4.1 Intestinal digestion and absorption function

Enteroendocrine cells (EECs) in the lining of the gut constitute the main part of the intestinal hormone pathway. They can release neuropeptides in response to chemical byproducts and signaling molecules released by intestinal bacteria. These neuropeptides can activate vagus sensory neurons or act directly on effector tissues through circulation. Moreover, the receptors of EEC hormones exist in the vagus nerve sensory neurons that innervate the gut. Through this pathway, they can control many physiological processes, such as gastric emptying, intestinal motility, insulin release, satiety and hunger, indicating that the intestinal flora can regulate physiological processes such as digestive function through neural pathways by affecting EEC. As mentioned above, SCFAs produced by the GM can promote intestinal endocrine cells to secrete GLP-1, PYY, GABA and 5-HT by binding to FFAR2 and FFAR3. Some of these substances act as neurotransmitters, and some can regulate the release of neurotransmitters, which can affect the contraction and relaxation of intestinal smooth muscle, thereby regulating the peristalsis and transport function of the intestine and maintaining the normal digestion and absorption process of the intestine (Silva et al., 2020).

### 4.2 Intestinal immune function

The GM and the ENS can interact with each other, and this interaction can also affect the function of intestinal immunity. The GM can regulate intestinal barrier function through the ENS, thereby affecting local intestinal immunity. Conversely, the GM and its metabolites can stimulate the ENS and promote the secretion of mucus by intestinal epithelial cells. The tight junction between epithelial cells is an extremely important part of the intestinal epithelial barrier (Gordon and Harbord, 2014). As a part of the physical barrier, the mucus layer can prevent direct contact between pathogens and intestinal epithelial cells and reduce the invasion of pathogens. The ENS can also regulate the expression and distribution of tight junction proteins between intestinal epithelial cells. For example, under the stimulation of bacterial flora, the ENS enhances the expression of tight junction proteins such as occludin and claudins through related signaling pathways, strengthening the connection between intestinal epithelial cells, preventing pathogens and harmful substances from entering the tissue through the intestinal epithelium, maintaining the integrity of the intestinal barrier, and protecting the local immune environment of the intestinal epithelium (Morais et al., 2021).

In addition, studies have shown that neurotransmitters released by enteric neurons can also regulate the migration, activation and differentiation of immune cells. In the regulation of the T helper cell 17/regulatory T cell (Th17/Treg) balance, some substances released by enteric neurons may promote the differentiation and function of Treg cells while inhibiting the production of Th17 cells. The interaction between the intestinal flora and the ENS indirectly affects the behavior of immune cells, regulates the Th17/Treg balance, and ultimately affects the intestinal immune environment to maintain intestinal immune homeostasis (Obata et al., 2020).

The causal direction of these interactions in humans is unclear, and extrapolating from germ-free or ENS-ablated animal models to the complex human intestinal immune system requires caution. Future studies should leverage human organoid–immune cell co-cultures to dissect ENS–immune crosstalk in a controlled yet human-relevant context.

### 4.3 Microbiota-ENS dominant mechanisms specific to digestive system diseases

#### 4.3.1 IBD: the vicious cycle centered on neuroimmune inflammation

In IBD, the microbiota–ENS axis operates as an “inflammation amplifier” —a self-perpetuating circuit in which microbial dysbiosis, ENS dysfunction, and neuroimmune activation mutually reinforce one another to sustain chronic intestinal inflammation. This amplification is orchestrated through at least two interconnected GABAergic mechanisms. Depletion of GABA-producing GM in ulcerative colitis patients leads to a significant reduction in luminal and tissue GABA levels (Aggarwal et al., 2017). This deficiency exerts dual pathological effects: first, loss of inhibitory tone in the ENS, where GABA deficiency disrupts the excitatory–inhibitory balance within enteric neural microcircuits, leading to dysmotility; second, release of immune brakes, as GABA normally exerts immunosuppressive effects—its absence allows excessive activation of the p38 MAPK signaling pathway in immune cells, triggering a pro-inflammatory cytokine storm (TNF-α, IL-1β). These cytokines not only directly damage the intestinal mucosa but also subsequently attack and remodel the ENS, inducing neuronal apoptosis and glial activation—thereby closing a destructive feedback loop. Concurrently, GABAergic neurons within the ENS release GABA to activate epithelial GABA receptors, inducing expression of insulin-like growth factor-binding protein 7 (Igfbp7). This paracrine signal sustains type 3 innate lymphoid cells (ILC3s), key immune cells that safeguard the mucosal barrier (Liu et al., 2025a). However, this protective mechanism is susceptible to disruption when ENS integrity is compromised by the inflammatory milieu.

Together, these findings reveal that GABAergic signaling serves as the pivotal bridge linking dysbiosis, ENS dysfunction, and disrupted immune homeostasis. Whether the initial insult stems from microbial GABA depletion or ENS-derived GABA dysregulation, the consequence is compromised barrier function and exacerbated inflammation. This mechanistic insight reframes IBD as a disorder of neuroimmune dialogue, suggesting that future therapeutic strategies should move beyond broad immunosuppression toward precision interventions that repair specific signaling pathways—such as GABAergic modulation—to break the amplification cycle.

While the slow neuro-immune loop dominates chronic inflammation, emerging evidence of bacterial galvanotaxis raises the question: do inflammation-associated changes in the gut’s bioelectric field cause pathobionts to locally congregate, creating persistent hotspots of microbial-ENS activation?

#### 4.3.2 IBS: multidimensional functional disorder centered on visceral hypersensitivity

In IBS, the microbiota–ENS axis functions as a “signal distorter” —amplifying, dampening, or misrouting neural signals to drive visceral hypersensitivity and dysmotility. This distortion arises from a triad of microbial influences on ENS function.

First, microbial metabolites directly modulate serotonergic signaling. Spore-forming bacteria promote 5-HT synthesis in ECCs; the resulting 5-HT elevation activates 5-HT3 receptors on sensory afferents, amplifying pain signals and contributing to abdominal pain and diarrhea (Mawe et al., 2006). Conversely, reduced Lactobacillus abundance lowers 5-HT levels, weakening propulsive signals and predisposing to constipation. Second, microbial influence extends to dopaminergic pathways. Gut bacteria modulate dopamine metabolism, and activation of dopamine D2 receptors attenuates visceral hyperalgesia (Strandwitz, 2018; Nozu et al., 2019), suggesting that microbiota can either amplify or dampen pain signals depending on the neurochemical context. Third, probiotics such as Saccharomyces boulardii directly alter myenteric neuron neurochemistry (Raskov et al., 2016), indicating that bacterial–neuronal interactions can reset distorted signaling patterns—a principle now being harnessed for therapeutic development.

These microbiota–ENS distortions manifest distinctly across IBS subtypes. In IBS-D, dysbiosis drives excitatory signal amplification, resulting in ENS-mediated hypersecretion and hypermotility. In IBS-C, dysbiosis leads to inhibitory signal dominance, impairing propulsive ENS function and delaying colonic transit. In visceral hypersensitivity-predominant IBS, dysbiosis triggers IL-6/TNF-α release and mast cell histamine activation, which stimulates TRPV1 channels on sensory neurons, distorting the gain of pain signaling (Chen et al., 2022).

The newly described neuropod cell-mediated glutamatergic signaling offers a mechanism for the millisecond-scale symptom onset after food intake in IBS patients, distinct from the slower (minute-scale) metabolite-driven pathways. We hypothesize that a ‘fast distortion’ in this synaptic route may underlie meal-related urgency and pain.

#### 4.3.3 CRC: microenvironment remodeling centered on tumor support

In CRC, the GM and ENS collaboratively establish a pro-tumor microenvironment, functioning as a “tumor accomplice” that actively promotes malignancy through metabolic crosstalk and neural reprogramming.

GM-derived metabolites drive cancer stem cell activation via ENS signaling. GM imbalance leads to abnormal elevation of specific SCFAs, such as isovaleric acid. Isovaleric acid directly stimulates ECCs and enteric neurons, prompting substantial 5-HT synthesis and release. Secreted 5-HT binds to receptors on CSCs, activating the canonical oncogenic Wnt/β-catenin signaling pathway. This cascade drives CSC self-renewal, proliferation, and tumor progression (Mawe and Hoffman, 2013; Zhu et al., 2022). This “isovaleric acid–5-HT–CSCs axis” reveals a novel mechanism whereby the microbiota directly regulates tumor-initiating cell fate via ENS neurotransmitters.

**Figure 4:**
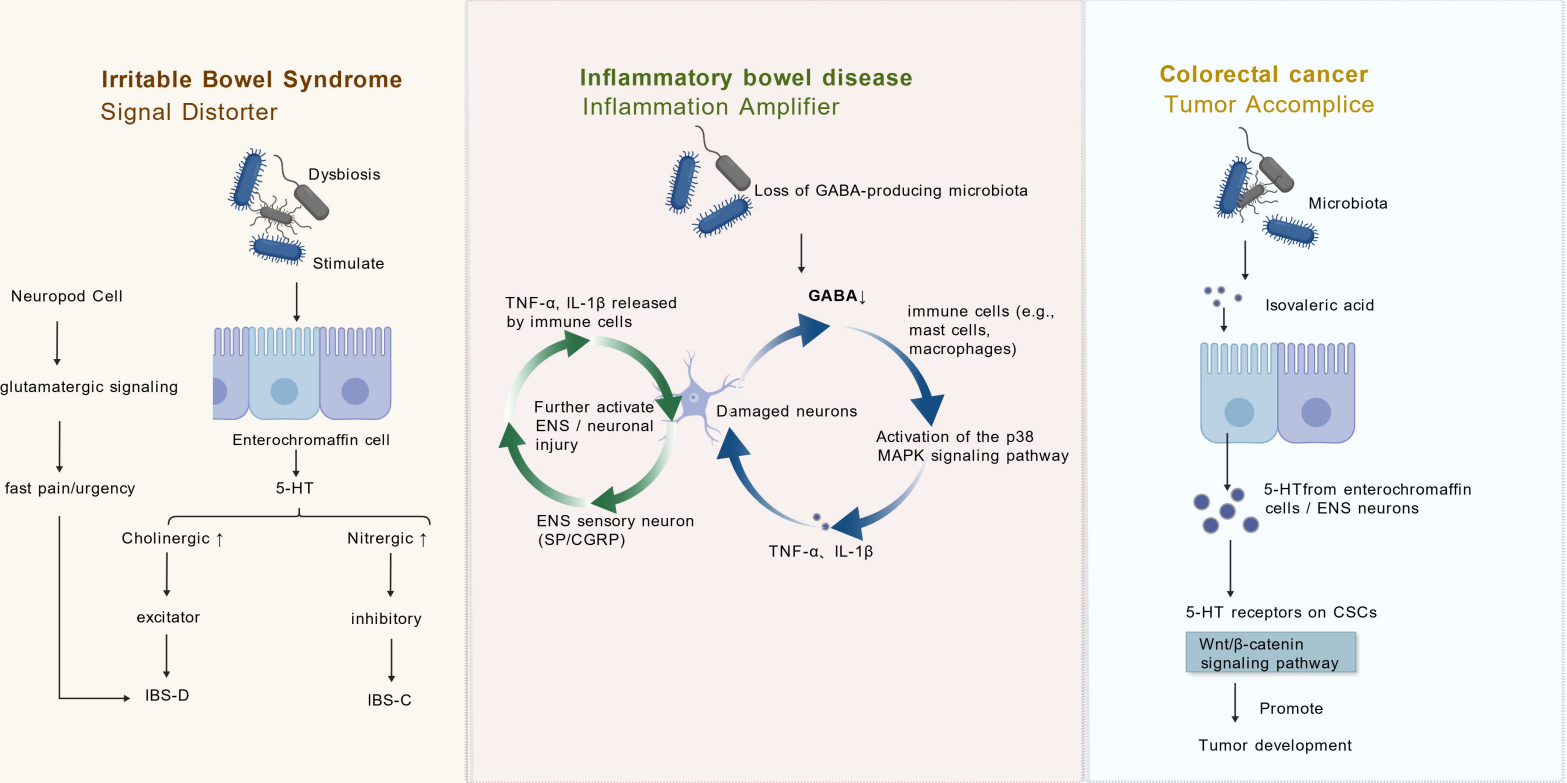
Disease-specific mechanisms of the microbiota-ENS axis. **(A)** IBS: Microbial signals trigger neuropod cell-mediated fast pain/urgency; dysbiosis drives 5-HT-mediated cholinergic/nitrergic imbalance. **(B)** IBD: GABA depletion relieves immunosuppression and activates p38 MAPK/TNF-α/IL-1β; ENS sensory neurons (SP/CGRP) amplify neuroimmune loop **(C)** CRC: Microbiota-derived isovaleric acid stimulatės 5-HT release, activating Wnt/β-catenin signaling and tumor progression. (Created with BioGDP.com).

The microbiota–ENS axis exhibits distinct functional dysregulation across different digestive disorders. (**Figure 4**) (Jiang et al., 2025)Understanding these disease-specific dominant mechanisms not only deepens pathophysiological insight but also enables precision medicine approaches.

### 4.4 An integrated mechanistic framework: testable hypotheses for the GM−ENS axis

Building on the multi-timescale signaling hierarchy introduced in Section 3.2.5, we now apply this framework to digestive diseases. We propose that IBS is primarily a disorder of fast (neuropod) and intermediate (metabolite/neurotransmitter) signals, leading to rapid symptom fluctuations. In contrast, IBD is dominated by slow (immune/plasticity) signals, resulting in a self-perpetuating amplifier. CRC exploits all layers, but critically co-opts the intermediate serotonergic pathway to remodel the stem cell niche.

To move beyond descriptive analogies and provide a strong, testable mechanistic framework, we synthesize the above disease−specific mechanisms into the following integrated hypotheses.

We propose that a positive feedback loop connects ENS sensory neurons with innate immune cells in IBD. Following dysbiosis and epithelial barrier disruption, ENS sensory neurons release substance P and CGRP, which stimulate mast cells and macrophages to produce TNF−α and IL−1β. These cytokines further sensitize enteric neurons and impair barrier integrity, perpetuating chronic inflammation. This loop can be tested by conditionally silencing ENS sensory neurons (e.g., Nav1.8^+^ or TRPV1^+^ neurons) in preclinical models. Predictions include reduced colonic TNF−α/IL−1β levels and attenuated histological inflammation upon neuronal silencing, and that pharmacological blockade of NK1R or IL−1β will break the cycle even without broad immunosuppression.

In IBS, we hypothesize that the ratio of cholinergic to nitrergic ENS activity is differentially skewed across subtypes: a relative cholinergic predominance in IBS−D and a relative nitrergic predominance in IBS−C. This ratio can be quantified in human colonic biopsies by the density of ChAT^+^ versus nNOS^+^ neurons or by measuring acetylcholine and nitric oxide metabolites. The hypothesis predicts that restoring this ratio to the normal range—for example, using a 5−HT_4_ agonist in IBS−C or a 5−HT_3_ antagonist in IBS−D—will normalize colonic transit and relieve the predominant bowel symptom, and that failure to correct the ratio will predict poor clinical response.

In CRC, we hypothesize that GM−derived branched−chain short−chain fatty acids, such as isovaleric acid, stimulate ECCs and enteric neurons to release 5−HT. This 5−HT acts on 5−HT_2_A or 5−HT_4_ receptors on CSCs, activating the Wnt/β−catenin pathway and driving CSCs self−renewal and tumor progression. The hypothesis can be tested in patient−derived CRC organoids or xenografts by manipulating isovaleric acid levels, blocking 5−HT synthesis (TPH1 inhibitor), or antagonizing 5−HT receptors. Predictions are that pharmacological inhibition of 5−HT signaling will reduce CSCs sphere formation and attenuate tumor growth, and that fecal isovaleric acid levels will correlate positively with tumor 5−HT content and poor prognosis.

Beyond the specific limitations already discussed in individual mechanistic sections, several cross−cutting integrative issues warrant emphasis. First, multiple mechanisms exhibit context−dependent or even contradictory effects in the literature (e.g., the bidirectional actions of short−chain fatty acids and GABA). Although these discrepancies have been noted in the respective subsections, their integrated significance across different disease contexts remains unclear. Second, in IBD, whether ENS activation is a driver or a consequence of intestinal inflammation remains unresolved — animal studies suggest bidirectional causality, but human prospective data are lacking. Similarly, in IBS, whether the observed shift in the excitatory/inhibitory balance is a cause or a consequence of microbial dysbiosis awaits longitudinal validation. Furthermore, technical bottlenecks are equally prominent: difficulty in obtaining longitudinal access to human ENS tissue, lack of non−invasive tools for assessing ENS function, and the inability of current animal models to fully recapitulate the heterogeneity of human disease collectively represent major obstacles for the field.

## 5 Therapeutic strategies targeting the GM–ENS axis

Therapeutic strategies targeting the GM-ENS axis can be categorized by their maturity: (i) established clinical interventions (dietary modification, rifaximin, TCAs, FMT) with documented efficacy in humans; (ii) emerging but evidence−limited approaches (probiotics, engineered biotherapeutics) where clinical translation is ongoing; and (iii) speculative/hypothesis−driven strategies (synthetic consortia, ENS−targeted neuromodulators, neurotransmitter−based antineoplastics) supported primarily by preclinical evidence.

### 5.1 Microbiota-directed interventions

#### 5.1.1 Dietary modification (established)

Diet strongly influences GM composition and function (Zhang et al., 2010; Lankelma et al., 2015), with distinct patterns differentially affecting the ENS. High-fat diets trigger inflammation and alter neuronal receptor sensitivity, leading to ENS “desensitization” and impaired motility. In contrast, high-fiber diets promote beneficial “remodeling”: microbiota ferment fiber into SCFAs (e.g., butyrate), which enhance excitatory neuron activity, induce neuroplasticity, and strengthen peristalsis (Soret et al., 2010). Fasting periodically elevates ghrelin, heightening ENS sensitivity to prokinetic signals and restoring normal response thresholds (Roosen et al., 2012).

These insights translate into practical dietary strategies. For ENS desensitization and dysmotility from high-fat diets: restrict saturated fat, increase omega−3 fatty acids to reduce inflammation and restore neuronal responsiveness. For functional constipation and hypomotility: adopt high−fiber diets to boost butyrate production and enhance ENS excitatory activity. For reduced ENS sensitivity: consider intermittent fasting to harness ghrelin peaks and “reset” ENS responsiveness. Integrating fat restriction, fiber supplementation, and fasting offers a multi−targeted approach to regulate ENS function and treat motility disorders at their source.

These dietary interventions can be reinterpreted through the mechanistic framework proposed in section 4.4. For IBS−C, where the hypothesis predicts a relative nitrergic (inhibitory) predominance, high−fiber diets enhance butyrate production, which we propose acts via FFAR3 on ChAT^+^ neurons to enhance cholinergic excitatory activity and restores the excitatory/inhibitory balance. For IBS−D, characterized by cholinergic overdrive, fat restriction reduces inflammation−induced neuronal hypersensitization, dampening excessive excitatory signals. Intermittent fasting, by elevating ghrelin, resets ENS response thresholds and may be particularly useful when both excitatory and inhibitory signals are distorted. Thus, dietary modifications are not merely supportive measures but mechanism−based interventions that correct specific ENS signaling imbalances.

#### 5.1.2 Probiotics and engineered biotherapeutics (emerging)

Probiotics—live microorganisms that confer health benefits—have been used in ENS−related disorders (Ji et al., 2025). They support ENS function indirectly by inhibiting pathogens, strengthening the intestinal barrier, and reducing inflammation.

However, traditional probiotics often fail due to poor survival and colonization in the harsh gut environment. Recent advances address this through material engineering and bioorthogonal chemistry. One innovative method coats Lactococcus lactis with a β−glucan−based shell (Lp@CGN). This coating provides three advantages: it boosts survival 276−fold in simulated gastrointestinal conditions, degrades specifically in the colon, and acts as a prebiotic—its breakdown products nourish beneficial bacteria. In ulcerative colitis models, this engineered probiotic alleviates disease by restoring barrier integrity, modulating inflammatory cytokines, correcting dysbiosis, and delivering sustained short−chain fatty acids. This work marks a shift from simple strain supplementation to programmable, functionally enhanced “living biotherapeutics.”

The therapeutic effects of probiotics can also be understood through disease−specific ENS mechanisms. In IBS, certain probiotics such as Saccharomyces boulardii have been reported to influence enteric neuron function. Based on the framework proposed in section 4.4, we speculate that such effects could reset the distorted excitatory/inhibitory ratio. In IBD, the same engineering strategies described above can be interpreted as breaking the neuro−immune amplification loop (section 4.3.1) by reducing the pro−inflammatory cytokines that sustain ENS activation. Future probiotic development should move beyond empirical strain selection toward mechanism−driven design – for example, engineering bacteria that produce specific GABAergic signals to restore inhibitory tone in IBD, or that degrade excessive 5−HT in IBS−D to rebalance serotonergic signaling.

#### 5.1.3 Precision antibiotics (established for rifaximin)

Antibiotics can transiently alter GM composition to eliminate harmful signals that persistently activate the ENS, creating a window for functional recovery. This “signal−based” rationale is exemplified by rifaximin, a non−absorbable antibiotic used in diarrhea−predominant IBS. By suppressing fermentative bacteria that produce gas and osmotically active metabolites, rifaximin reduces the physical and chemical stimuli driving ENS hyperexcitability (Lacy et al., 2021). More importantly, transient microbial resetting allows the distorted excitatory/inhibitory balance to normalize – which explains why clinical benefits often outlast treatment duration. Future precision antibiotic strategies should move beyond empirical use toward biomarker−guided selection (e.g., identifying patients with overgrowth of specific gas−producing or 5−HT−modulating bacteria).

#### 5.1.4 FMT (established for C. difficile)

FMT involves transferring fecal microorganisms from a healthy donor to a patient, rapidly restoring gut microbial diversity and function (Ooijevaar et al., 2019). Initially developed for recurrent Clostridium difficile infection (Gough et al., 2011; McDonald et al., 2018), its use has now expanded to IBS and IBD—conditions closely linked to ENS dysfunction.

From the perspective of the GM−ENS axis, FMT can be viewed as a multi−targeted intervention that simultaneously addresses the three pillars of signaling: microbial metabolites (e.g., restoring butyrate to modulate ENS excitability), neuroactive substances (e.g., replenishing GABA to restore inhibitory tone), and immune mediators (e.g., reducing pro−inflammatory cytokines that drive neuroinflammation). Additionally, competitive exclusion of pathobionts removes sources of neuroactive toxins and pro−inflammatory stimuli at their origin. In line with the hypotheses from section 4.4, successful FMT in IBS would be expected to restore the cholinergic/nitrergic balance, while in IBD it would break the neuro−immune amplification loop. However, the inconsistent outcomes of FMT in current trials highlight the need for biomarker−guided donor selection – for example, matching donors with high butyrate or GABA production capacity to patients with corresponding deficiencies. Until such mechanistic stratification becomes routine, FMT will remain a broad, imprecise tool rather than a precision therapy.

### 5.2 Host nervous system−directed interventions

These strategies directly target neural components of the GM–ENS axis to alleviate symptoms.

#### 5.2.1 Antidepressants (established)

Tricyclic antidepressants (TCAs) are effective in IBS, particularly in reducing abdominal pain and diarrhea (Temido et al., 2025). They modulate serotonergic (5−HT) and noradrenergic signaling in both the central nervous system and the ENS, raising pain thresholds, regulating motility, and potentially stabilizing ENS electrical activity. Nevertheless, TCAs act as broad neuromodulators without significant ENS subtype selectivity. Their clinical utility therefore supports the concept of neuromodulation in IBS but also highlights the need for more precise, peripherally restricted agents that can correct specific ENS signaling imbalances without systemic side effects. In this regard, newer peripherally acting neuromodulators or ENS−targeted receptor agonists (e.g., 5−HT_4_ agonists for constipation) represent a more mechanism−driven approach.

#### 5.2.2 Neurotransmitter−targeted antineoplastic therapy (speculative)

As proposed in section 4.4, in CRC, microbiota−derived metabolites (e.g., isovaleric acid) stimulate ECCs and ENS neurons to release 5−HT, which then activates Wnt/β−catenin signaling in cancer stem cells via 5−HT receptors. This model offers precise therapeutic entry points. Therefore, blocking any node of this axis – using a gut−restricted TPH1 inhibitor (e.g., telotristat ethyl) to reduce peripheral 5−HT synthesis, 5−HT_2_A/4 receptor antagonists, or downstream Wnt/β−catenin inhibitors – may suppress CRC stem cell self−renewal and tumor progression. This represents a mechanism−based precision strategy targeting neuro−microbial crosstalk in the tumor microenvironment, rather than broad cytotoxics.

Neurotransmitter signaling is an emerging anticancer target, with potential applications in immunotherapy through receptor modulation and tumor microenvironment interference (Leung et al., 2023). **Table 1** summarizes neurotransmitter–gastrointestinal tumor relationships and potential therapeutic targets.

**Table 1:** Relationships between neurotransmitters and digestive tract tumors.

Neurotransmitters	Association with tumors of the digestive tract	Reference
Acetylcholine (ACh)	Cholinergic nerve cells release acetylcholine (ACh) and activate M3/M4 muscarinic receptors on the surface of tumor cells, triggering phosphorylation of downstream ERK and STAT3 signaling pathways. STAT3 can upregulate the expression of pro-tumor genes (such as Cyclin D1 and Bcl-2), accelerate cell cycle progression and inhibit apoptosis. Moreover, ERK pathway promotes tumor invasion and metastasis by enhancing MMP-9 secretion.	(Hernandez et al., 2025)
Dopamine	High expression of dopamine receptor 1 (DRD1) stimulates the malignant activity of hepatocellular carcinoma by regulating the cAMP/PI3K/AKT/CREB pathway, leading to poor patient. Treatment with DRD1 specific antagonist SCH23390 significantly inhibits cell invasion and migration and tumor growth *in vivo*. Upregulation of dopamine receptor 2 (DRD2) expression is associated with increased malignant potential of gastric cancer and PDAC.	(Yan et al., 2020)
Catecholamines	β2 receptors can promote the development of liver cancer by inhibiting the degradation of hypoxia-inducible factor 1α (HIF1α). Catecholamines act directly on the β receptors on tumor cells to stimulate the survival, proliferation and metastasis of *de novo* tumors. Activation of α2 receptors can enhance the activation of CD4+/CD8+ T cells by directly acting on macrophages and induce a strong anti-tumor immune response.	(Wu et al., 2016; Zahalka et al., 2017)
5-hydroxytryptamine	5-HT produced by serotonergic neurons in the intestines initiates the process of self-renewal and tumorigenesis of CRC stem cells. Serotonin accumulation has been observed in p53-driven models of pancreatic cancer and is associated with accelerated growth of pancreatic tumors.	(Jiang et al., 2017; Zhu et al., 2022)
Glycine	Glycine is a major inhibitory neurotransmitter in the brain that exerts inhibitory effects by binding to specific postsynaptic receptors and opening chloride channels inside the receptors. Studies have shown that when glycine is added to the culture conditions of various tumor cell lines, the growth of tumor cells is significantly increased.	(Jain et al., 2012)
γ-aminobutyric acid	γ-aminobutyric acid type A receptor (GABA AR) signaling can lead to tumor growth and invasion, while γ-aminobutyric acid type B receptor (GABA BR) signaling, conversely, can significantly inhibit the growth of tumor cells and promote their apoptosis. The mechanism is related to AKT signaling, CREB signaling and the tyrosine kinase receptor.	(Schuller et al., 2008; Huang et al., 2023)
Nerve growth Factor (NGF)	Overexpression of NGF may promote the survival and motility of hepatocellular carcinoma cells through the tropomyosin receptor kinase A (TrkA) pathway. Similar effects of the NGF-TrkA pathway have been observed in patients with other GI tumors, such as colorectal and pancreatic cancers	(Blondy et al., 2019)

### 5.3 Immune−mediated interventions (speculative)

Based on the “inflammation amplifier” model proposed in section 4.4 – where ENS sensory neurons release substance P and CGRP to activate innate immune cells, and the resulting TNF−α/IL−1β further sensitize ENS neurons – we hypothesize that blocking specific nodes of this positive feedback loop may be more effective than general immunosuppression. Promising strategies include NK1R antagonists (blocking substance P) and locally delivered IL−1β neutralizing antibodies. Although still in early stages, such mechanism−driven immune interventions offer a path toward precision therapy for IBD patients with ENS hyperexcitability. Preclinical studies support this concept: NK1R antagonists and IL-1β blockade reduce colitis severity in animal models (Stucchi et al., 2000), though clinical trials in unselected IBD patients have shown mixed results, highlighting the need for biomarker-guided patient selection.

## 6 Conclusions

In conclusion, the GM-ENS axis is not merely a passive relay but the primary peripheral architect of gut homeostasis and disease. By moving beyond descriptive analogies to propose testable, disease-specific mechanistic frameworks—the ‘signal distortion’ (IBS), ‘inflammation amplifier’ (IBD), and ‘tumor accomplice’ (CRC)—we provide a roadmap for future research. Crucially, integrating fast neuropod, intermediate bioelectric, and slow immune signals into a multi-timescale hierarchy transforms our understanding from a static network to a dynamic control system. The path forward lies in dissecting how these layers interact in space and time, and then designing circuit-specific, mechanism-based interventions that repair, rather than merely manage, GM-ENS dysfunction.

## 7 Outlook and future directions

Future research should focus on deciphering the bidirectional regulatory network between the gut microbiome and the ENS and accelerating its translation into diagnostic and therapeutic applications for digestive disorders.

First, developing dynamic perturbation–observation research paradigms. Most current studies rely on static correlations or endpoint indicators. Future research should adopt techniques such as optogenetics, chemogenetics, and real-time *in vivo* imaging to capture how microbial signals are dynamically sensed and integrated by the ENS across physiologically relevant timescales. Moving from correlation to causal mechanistic elucidation is essential for identifying actionable therapeutic targets.

Second, establishing microbiome-based biomarker panels for precision medicine. Current symptom-based classifications (e.g., for IBS) exhibit significant heterogeneity. Multidimensional biomarker models that integrate specific microbial taxa, neuroactive metabolites (e.g., secondary bile acids, SCFAs), and host ENS-related gene expression profiles could define mechanism-based disease subtypes. For instance, a signature combining Bacteroides enrichment, low butyrate levels, and reduced expression of cholinergic neuronal markers could identify a novel “dysmotility-type” IBS subtype amenable to targeted prokinetic therapy.

Third, advancing targeted microbiota intervention strategies. While interventions such as probiotics and FMT show promise, inconsistent results and safety concerns persist. The field should shift toward biomarker-guided, pathway-specific precision strategies. Postbiotics—defined microbial metabolites such as butyrate or tryptophan derivatives—offer advantages in composition consistency, quality control, and safety. Synthetic microbial consortia and phage therapy represent additional avenues for precisely remodeling dysbiotic communities.

Fourth, exploring applications in gastrointestinal oncology. Beyond traditional microbiome–immune interactions, the GM–ENS axis plays a critical role in tumor progression. Precisely modulating tumor-associated microbiota or ENS-derived signals may delay malignancy through microenvironment remodeling. This strategy could extend across gastrointestinal tumors, including gastric and pancreatic cancers, potentially establishing a new paradigm for cancer microenvironment regulation. Nevertheless, translating these prospects into clinical reality faces substantial hurdles: the difficulty of accessing human ENS tissue for longitudinal monitoring, the challenge of distinguishing causal microbial signals from bystander effects, and the need for better preclinical models that recapitulate human disease heterogeneity. Addressing these limitations will require interdisciplinary collaboration and iterative validation between bench and bedside.

Fifth, prioritizing the ENS as a direct therapeutic target. While central nervous system (CNS)-directed interventions (e.g., antidepressants, neuromodulators) can relieve symptoms via the brain-gut axis, they often carry systemic side effects – including sedation, weight gain, and cognitive impairment – due to broad receptor distribution. In contrast, the ENS operates as a semi-autonomous peripheral network. Targeting its local neural circuits, glial cells, or receptor subtypes (e.g., 5-HT4 receptors on enteric neurons, GABAergic signaling within myenteric plexuses) promises greater mechanistic directness and fewer off-target effects. Emerging ENS-focused strategies include: (i) regionally restricted prokinetics (e.g., prucalopride) that enhance colonic transit without crossing the blood-brain barrier; (ii) glial modulators that break neuroinflammation cycles in IBD; and (iii) ultrasound-based peripheral neuromodulation to reset dysmotility patterns. By anchoring future precision therapies to the GM-ENS axis, we can bypass the “central bottleneck” and achieve durable disease modification with improved safety profiles.

In summary, the path forward lies in establishing a closed-loop system from microbiome-based diagnostics to precision intervention: identifying patient-specific dysbiosis patterns via biomarkers, then selecting the most appropriate intervention—whether multi-strain live biotherapeutics, defined postbiotics, or novel drugs targeting downstream pathways—to achieve disease modification in ENS-related digestive disorders.
